# Supplementary material for: An open-source framework for large-scale, flexible evaluation of biomedical text mining systems
Source: J Biomed Discov Collab. 2008 Jan 29;3:1. doi: 10.1186/1747-5333-3-1 (PMC2276192; doi:10.1186/1747-5333-3-1)
Supplement: Additional file 1 — Evaluation platform source code. The additional file contains the software evaluation framework discussed in this paper. The most current release can be downloaded from SourceForge.net [1]. See the accompanying README file for instructions on installation and use. Also included in the distribution is a collection of UIMA wrappers for some commonly used BioNLP tools and annotated corpora. [file 1747-5333-3-1-S1.zip › bionlp_uima_repository-1.0.1/docs/api/src-html/edu/uchsc/ccp/util/nlp/GenericTextUtils.html]

```
001    /*
002     *
003     * The Original Code is UCHSC Utilities.
004     *
005     * The Initial Developer of the Original Code is University of Colorado.  
006     * Copyright (C) 2005.  All Rights Reserved.
007     *
008     * UCHSC Utilities was developed by the Center for Computational Pharmacology
009     * (http://compbio.uchsc.edu) at the University of Colorado Health 
010     *  Sciences Center School of Medicine with support from the National 
011     *  Library of Medicine.  
012     *
013     *
014     * Contributor(s):
015     *   William A. Baumgartner <william.baumgartner@uchsc.edu> (Original Author)
016     *   
017     * Created: August 2005
018     * Dependencies:
019     *              edu.uchsc.ccp.util.RunExecutable
020     * ToDo: 
021     *              tokenizeIntoWords should return an arraylist of int[]'s not String[]'s
022     *              determineDocumentSection can probably be deprecated since the new UIMA type system includes a documentSection annotation
023     * Changes:
024     *              01/14/06 - wab - added openPrintStream(outputFileLocation, append)
025     */
026    package edu.uchsc.ccp.util.nlp;
027    
028    import java.io.BufferedOutputStream;
029    import java.io.BufferedReader;
030    import java.io.File;
031    import java.io.FileInputStream;
032    import java.io.FileOutputStream;
033    import java.io.IOException;
034    import java.io.InputStreamReader;
035    import java.io.PrintStream;
036    import java.util.ArrayList;
037    import java.util.Arrays;
038    import java.util.Collections;
039    import java.util.HashMap;
040    import java.util.HashSet;
041    import java.util.List;
042    import java.util.Map;
043    import java.util.Set;
044    import java.util.regex.Matcher;
045    import java.util.regex.Pattern;
046    import java.util.regex.PatternSyntaxException;
047    
048    import javax.xml.parsers.SAXParser;
049    import javax.xml.parsers.SAXParserFactory;
050    
051    import org.xml.sax.XMLReader;
052    
053    import edu.uchsc.ccp.util.RunExecutable;
054    import edu.uchsc.ccp.util.nlp.annotation.AnnotationSet;
055    import edu.uchsc.ccp.util.nlp.annotation.Annotator;
056    import edu.uchsc.ccp.util.nlp.annotation.TextAnnotation;
057    import edu.uchsc.ccp.util.nlp.mention.ClassMention;
058    import edu.uchsc.ccp.util.nlp.mention.ClassMentionTypes;
059    
060    /**
061     * This class encompasses a collection of utilities for processing and dealing with text.
062     * 
063     * @author William A. Baumgartner, Jr.
064     * 
065     */
066    public class GenericTextUtils {
067        private final static boolean DEBUG = false;
068    
069        /**
070         * Opens a PrintStream for printing to the given output file location
071         * 
072         * @param outputFileLocation
073         *            path to the output file
074         * @return an open PrintStream
075         * @throws IOException
076         */
077        public static PrintStream openPrintStream(String outputFileLocation) throws IOException {
078            return new PrintStream(new BufferedOutputStream(new FileOutputStream(outputFileLocation)));
079        }
080    
081        /**
082         * Opens a PrintStream for printing to the given output file location
083         * 
084         * @param outputFileLocation
085         *            path to the output file
086         * @param append
087         * @return an open PrintStream
088         * @throws IOException
089         */
090        public static PrintStream openPrintStream(String outputFileLocation, boolean append) throws IOException {
091            return new PrintStream(new BufferedOutputStream(new FileOutputStream(outputFileLocation, append)));
092        }
093    
094        /**
095         * Opens a BufferedReader for reading from a text file
096         * 
097         * @param inputFileLocation
098         *            path to the input file
099         * @return an open BufferedReader
100         * @throws IOException
101         */
102        public static BufferedReader openReader(String inputFileLocation) throws IOException {
103            return new BufferedReader(new InputStreamReader(new FileInputStream(inputFileLocation)));
104        }
105    
106        /**
107         * Returns some sample text
108         * 
109         * @return a string of sample text
110         */
111        public static String exampleDocText() {
112            return "I do not like Green Eggs and Ham. I do not like them Sam, I am. I do not like them here or there. I do not like them anywhere. I do not like them in a boat. I would not, could not, with a goat. I will not eat them in the rain. I do not like them on a train. I do not like them in a box. I will not eat them with a fox. I do not like them in a house. I would not, could not, with a mouse. I do not like Green Eggs and Ham. I do not like them. Sam, I am. ";
113        }
114    
115        /**
116         * Sorts a Hashmap by the values contained in the HashMap
117         * 
118         * @param inputMap
119         *            the HashMap to be sorted
120         * @return an ArrayList containing sorted KeyValuePairs
121         */
122        public static ArrayList<KeyValuePair> sortHashMapByValues(Map<String, Integer> inputMap) {
123    
124            ArrayList mapValues = new ArrayList<Integer>(inputMap.values());
125            ArrayList mapKeys = new ArrayList<String>(inputMap.keySet());
126    
127            inputMap = new HashMap();
128            ArrayList<KeyValuePair> kvlist = new ArrayList<KeyValuePair>();
129    
130            while (mapValues.size() > 0) {
131                KeyValuePair kvp = new KeyValuePair((String) mapKeys.get(0), ((Integer) mapValues.get(0)).intValue());
132                kvlist.add(kvp);
133                mapValues.remove(0);
134                mapKeys.remove(0);
135            }
136    
137            Collections.sort(kvlist);
138            return kvlist;
139        }
140    
141        /**
142         * This method performs the reverse of the Set.toString() method. It reads in a string with format "[A,B,C]" and returns a Set<String> with A, B,
143         * and C as members of the set.<br>
144         * <br>
145         * NOTE: If the original set contained Strings with commas, then this method will be unreliable.
146         * 
147         * @param setString
148         * @return
149         */
150        public static Set<String> getSetFromString(String setString) {
151            Set<String> returnSet = new HashSet<String>();
152    
153            /* remove the leading and trailing square brackets */
154            setString = setString.substring(1, setString.length() - 1);
155            String[] toks = setString.split(",");
156            for (String tok : toks) {
157                returnSet.add(tok.trim());
158            }
159            return returnSet;
160        }
161    
162        public static String[] STOPWORDS = { "i", "a", "is", "if", "be", "mm", "of", "no", "so", "mg", "ml", "as", "we", "at", "by", "in",
163                "on", "it", "to", "do", "kg", "km", "an", "did", "due", "any", "for", "use", "may", "and", "etc", "are", "but", "can", "our",
164                "how", "nor", "the", "has", "was", "had", "its", "all", "this", "done", "just", "also", "make", "upon", "show", "used", "very",
165                "from", "most", "were", "must", "some", "what", "have", "than", "here", "that", "such", "when", "been", "with", "both", "them",
166                "then", "into", "seem", "thus", "each", "made", "seen", "does", "they", "these", "about", "quite", "again", "which", "found",
167                "might", "shown", "using", "among", "those", "shows", "since", "while", "being", "their", "often", "would", "there", "could",
168                "during", "nearly", "having", "mostly", "enough", "always", "either", "mainly", "should", "showed", "before", "within",
169                "theirs", "itself", "rather", "really", "almost", "perhaps", "through", "several", "another", "various", "further", "because",
170                "neither", "between", "however", "without", "overall", "obtained", "although", "therefore", "regarding", "especially",
171                "significantly" };
172    
173        private static List<String> STOPWORDLIST = Arrays.asList(STOPWORDS);
174    
175        /**
176         * Removes stop words from the input text string. Stop words list taken from PUBMED.
177         * 
178         * @param queryStr
179         *            the string to be processed
180         * @return the input string without stop words
181         */
182        public static String removeStopWords(String queryStr) {
183    
184            
185            
186            for (int i = 0; i < STOPWORDS.length; i++) {
187                // if (queryStr.indexOf(" " + STOPWORDS[i] + " ") > -1) {
188                // System.out.println("Found stopword: " + stopwords[i]);
189                queryStr = queryStr.replaceAll(("(^| )(" + STOPWORDS[i] + ")($| )"), " ");
190                // }
191            }
192    
193            return queryStr;
194        }
195    
196        public static boolean isStopWord(String word) {
197            if (STOPWORDLIST.contains(word.toLowerCase())) {
198                return true;
199            } else {
200                return false;
201            }
202        }
203    
204        // public static List<TextAnnotation> tokenizeIntoSentences(String baseDirectory, String documentText) throws NoSuchMethodException {
205        // }
206    
207        /**
208         * This method tokenizes the input text into sentences via the KeX sentence tokenizing utility.<br>
209         * <br>
210         * It should be noted that sometimes the KeX sentence tokenizer modifies the output text (usually by adding or deleting a space) so that it is not
211         * possible to determine the offset in the original text. When this occurs an error is displayed to the screen.
212         * 
213         * @param baseDirectory -
214         *            location of oneline program (part of KeX Entity Identifier)
215         * @param documentText -
216         *            text to be tokenized into sentences (and words are delimited by whitespace)
217         * @return an ArrayList containing two ArrayLists, the first containing Strings where each String is a sentence, and the second containing Strings
218         *         where each String is a word (token).
219         * 
220         */
221        public static List<TextAnnotation> tokenizeIntoSentences(String baseDirectory, String documentText, String documentID) {
222    
223            // System.err.println("Tokenizing into sentences...");
224    
225            final Annotator annotator = new Annotator(3, "KeX", "KeX", "Fukada");
226    
227            final String outputFile = "sentenceTok.out";
228            final String inputFile = "sentenceTok.in";
229            final String execFilename = "sentTok_exec.sh";
230    
231            final String workDirectory = baseDirectory + "/work";
232            List<TextAnnotation> sentences = new ArrayList<TextAnnotation>();
233            // List<TextAnnotation> words = new ArrayList<TextAnnotation>();
234    
235            try {
236                PrintStream execFileStream = new PrintStream(new BufferedOutputStream(new FileOutputStream(baseDirectory + "/" + execFilename)));
237    
238                // load string into an input file
239                PrintStream inputFileStream = new PrintStream(new BufferedOutputStream(new FileOutputStream(workDirectory + "/" + inputFile)));
240                inputFileStream.println(documentText);
241                inputFileStream.close();
242    
243                // delete output file if it exists
244                File f = new File(workDirectory + "/" + outputFile);
245                if (f.exists()) {
246                    f.delete();
247                }
248    
249                // create execution script
250                // KeX requires a system variable: KEX to be set
251                execFileStream.println("export KEX=" + baseDirectory + ";");
252    
253                // KeX requires . to be in the path
254                execFileStream.println("export PATH=$PATH:.;");
255    
256                // change to the base directory
257                execFileStream.println("cd " + baseDirectory + "/bin;");
258    
259                // execute KeX
260                execFileStream.println("./oneline " + workDirectory + "/" + inputFile + " > " + workDirectory + "/" + outputFile + ";");
261    
262                execFileStream.close();
263    
264                // make execFile executable
265                RunExecutable.run("chmod 755 " + baseDirectory + "/" + execFilename, true);
266    
267                // run tokenizer
268                System.out.print("running Sentence Tokenizer...");
269                RunExecutable.run(baseDirectory + "/" + execFilename, true);
270                // System.out.println("complete.");
271    
272                // get sentences from output file, load them into sentences
273                // ArrayList
274                BufferedReader br = new BufferedReader(new InputStreamReader(new FileInputStream(workDirectory + "/" + outputFile)));
275    
276                // System.err.println("Reading KeX output file...");
277    
278                String line;
279                int sent_startLookingAtIndex = 0;
280                while ((line = br.readLine()) != null) {
281                    /** KeX sometimes adds in underscores such as between in_vitro.. we remove these in order to match the original text */
282                    line = line.replaceAll("_", " ");
283                    line = line.replaceAll("\\.", ". ");
284                    // String[] span = GenericTextUtils.findSpan(line, " " + line + " ", documentText, sent_startLookingAtIndex);
285    
286                    // System.err.println("Getting annotations... start looking at: " + sent_startLookingAtIndex);
287                    // System.err.println("Line: " + line);
288                    // System.err.println("DocumentText: " + documentText);
289    
290                    if (line.trim().length() > 0) {
291                        List<TextAnnotation> sentenceAnnots = GenericTextUtils.findTargetInText(line, documentText, sent_startLookingAtIndex,
292                                documentID, ClassMentionTypes.SENTENCE, annotator, false);
293    
294                        if (sentenceAnnots.size() > 0) {
295                            sent_startLookingAtIndex += sentenceAnnots.get(0).getCoveredText().length();
296    
297                            while (sentenceAnnots.get(0).getCoveredText().endsWith(" ")) {
298                                sentenceAnnots.get(0).setAnnotationSpanEnd(sentenceAnnots.get(0).getAnnotationSpanEnd() - 1);
299                                sentenceAnnots.get(0).setCoveredText(
300                                        sentenceAnnots.get(0).getCoveredText()
301                                                .substring(0, sentenceAnnots.get(0).getCoveredText().length() - 1));
302                            }
303                            sentences.addAll(sentenceAnnots);
304                        }
305                    }
306    
307                    // if (span[2] != null) {
308                    // sentences.add(span);
309                    // if (DEBUG) {
310                    // System.out.println("************************ FOUND SENTENCE: " + span[0] + " -- " + span[1] + "\t" + span[2]);
311                    // }
312                    // ArrayList wordtoks = GenericTextUtils.tokenizeIntoWords(line, span[2], Integer.parseInt(span[0]));
313                    // for (Iterator iterator = wordtoks.iterator(); iterator.hasNext();) {
314                    // String[] word_span = (String[]) iterator.next();
315                    // // System.out.println("WORD: " + word_span[0] + " -- " +
316                    // // word_span[1] + "\t" + word_span[2]);
317                    // words.add(word_span);
318                    // }
319                    // sent_startLookingAtIndex = Integer.parseInt(span[1]);
320                    // } else {
321                    // System.err.println("******************** MISSED SENTENCE: " + line);
322                    // }
323                }
324    
325                br.close();
326    
327            } catch (IOException ioe) {
328                System.err.println("IOException while creating kex_exec.sh file in KeXEISystem...");
329                ioe.printStackTrace();
330            }
331    
332            // ArrayList retArray = new ArrayList();
333            // retArray.add(sentences);
334            // retArray.add(words);
335            return sentences;
336        }
337    
338        // /**
339        // * This simple method tokenizes an input text into words based solely on whitespace and returns not only the words, but their corresponding
340        // spans
341        // * as well.
342        // *
343        // * @param tokenizedSentence
344        // * the text to be tokenized by whitespace (probably should have punctuation tokenized already)
345        // * @param originalText
346        // * the original (untokenized) text, used to compute word spans
347        // * @param docStartIndex
348        // * the offset for the beginning of the original text
349        // * @return an ArrayList of String arrays containing the spans for each token {span start, span end}
350        // */
351        //
352        // public static ArrayList tokenizeIntoWords(String tokenizedSentence, String originalText, int docStartIndex) {
353        // // System.out.println("DOCSTARTINDEX = " + docStartIndex);
354        // ArrayList words = new ArrayList();
355        // String[] toks = tokenizedSentence.split("\\s+");
356        // int word_startLookingAtIndex = 0;
357        // for (int i = 0; i < toks.length; i++) {
358        // // System.out.println("LOOKING FOR TOKEN: " + toks[i]);
359        // String[] span = GenericTextUtils.findSpan(toks[i], toks[i], originalText, word_startLookingAtIndex);
360        // // System.out.println("SPAN: " + span[0] + " -- " + span[1] + "\t" +
361        // // span[2]);
362        // word_startLookingAtIndex = Integer.parseInt(span[1]);
363        // span[0] = Integer.toString(Integer.parseInt(span[0]) + docStartIndex);
364        // span[1] = Integer.toString(Integer.parseInt(span[1]) + docStartIndex);
365        // words.add(span);
366        // }
367        // return words;
368        // }
369    
370        /**
371         * Determines the document section identifier for a given span based on the document section IDs and span lengths provided.
372         * 
373         * @param spanStart
374         *            the start of the span for the text whose document section ID is to be determined
375         * @param sectionIDs
376         *            an int[] containing the document section IDs associated with a given document
377         * @param sectionLengths
378         *            an int[] containing the lengths of each document section associated with a given document
379         * @return The document section ID.
380         */
381        public static int determineDocumentSection(int spanStart, int[] sectionIDs, int[] sectionLengths) {
382    
383            if (DEBUG) {
384                for (int i = 0; i < sectionIDs.length; i++) {
385                    System.out.println("----- SECID: " + sectionIDs[i] + "(" + sectionLengths[i] + ")");
386                }
387            }
388    
389            // determine document section
390            int documentSectionIDIndex = 0;
391            try {
392                int cumulativeLength = -1;
393                for (int j = 0; j < sectionIDs.length; j++) {
394                    if (spanStart > cumulativeLength) {
395                        documentSectionIDIndex = j; // sectionIDs.get(j);
396                        cumulativeLength += sectionLengths[j];
397                        if (DEBUG) {
398                            System.out.println("Span begin: " + spanStart + " < " + sectionLengths[j] + " = "
399                                    + sectionIDs[documentSectionIDIndex]);
400                        }
401                    }
402                }
403                return sectionIDs[documentSectionIDIndex];
404            } catch (NumberFormatException nfe) {
405                System.err.println("Number Format Exception while determining document section...");
406                System.err.println("span start = " + spanStart);
407                return -1;
408            }
409    
410        }
411    
412        /**
413         * Prepares a string to be used in a Regular Expression search. This method places a backslash in front of characters that need to be escaped in
414         * order to be recognized in a regular expression
415         * 
416         * @param regex
417         *            the String to be converted into a Regular Expression search string
418         * @return the converted String
419         */
420        public static String escapeCharacterForRegEx(String regex) {
421            final String[] escapedChars = { "\\", "(", ")", "{", "}", "+", "*", "?", "[", "]", "^", "$", ".", "|" };
422            final String escape = "\\";
423            int splitIndex = -1;
424            String tmpStr = "";
425            for (int i = 0; i < escapedChars.length; i++) {
426                // escape characters for substr
427                if ((splitIndex = regex.indexOf(escapedChars[i])) > -1) {
428                    if (DEBUG) {
429                        System.out.println("==== Found: " + escapedChars[i] + " in regex text  ====");
430                    }
431                    int fromIndex = 0;
432                    while (splitIndex > -1) { // check for mulitiple of same escapedChar in regex
433                        // insert \ in front of the escaped character
434                        tmpStr = regex.substring(0, splitIndex) + escape + regex.substring(splitIndex);
435                        regex = tmpStr;
436                        fromIndex = splitIndex + 2;
437                        splitIndex = regex.indexOf(escapedChars[i], fromIndex);
438                    }
439                }
440            }
441            return regex;
442        }
443    
444        // /**
445        // * This method is a utility for finding the span of a particular piece of text within the original document. The process works by
446        // finding a larger neighborhood of text within the original document, then the span of interest within the neighborhood. The procedure
447        // is designed to prevent errors due to multiple mentions of the same text within a document.
448        // * @param subStr the text for which a span is desired
449        // * @param neighborhood a neighborhood of text surrounding the subStr
450        // * @param wholeStr the original document
451        // * @param startLookingAtIndex the index within the original document to begin looking for the neighborhood text
452        // * @return String array of size three; where String[0] = span start, String[1] = span end, String[2] = covered text
453        // */
454        // public static String[] findSpan(String subStr, String neighborhood, String wholeStr, int startLookingAtIndex) {
455        // // create a regular expression for each gene - tok by space, then add
456        // // //s? in between tokens
457        // // this should work for all genes except those that appear >1 time in
458        // // the text
459        // // solution, keep track of index from where to start looking for
460        // // matches.
461        //
462        // // neighborhood is used to find the general position of the substring in
463        // // the whole string
464        // // the neighborhood should consist of ~5 words to ensure uniqueness
465        //
466        // // returns span[0]: start index; span[1]: end index; span[2]: covered
467        // // text
468        //
469        // if (DEBUG) {
470        // System.out.println("UNprocessed substr: " + subStr);
471        // System.out.println("UNProcessed neighborhood: " + neighborhood);
472        // }
473        //
474        // String[] span = new String[3];
475        // //String escapedChars = "(){}+*?[]^$.\\";
476        // String[] escapedChars = { "\\", "(", ")", "{", "}", "+", "*", "?", "[", "]", "^", "$", "." };
477        // String escape = "\\";
478        // int splitIndex = -1;
479        // String tmpStr = "";
480        // for (int i = 0; i < escapedChars.length; i++) {
481        // // escape characters for substr
482        // if ((splitIndex = subStr.indexOf(escapedChars[i])) > -1) {
483        // if (DEBUG) {
484        // System.out.println("==== Found: " + escapedChars[i] + " in substr text ====");
485        // }
486        // int fromIndex = 0;
487        // while (splitIndex > -1) { // check for mulitiple of same
488        // // escapedChar in substr
489        // // insert \ in front of the escaped character
490        // tmpStr = subStr.substring(0, splitIndex) + escape + subStr.substring(splitIndex);
491        // subStr = tmpStr;
492        // fromIndex = splitIndex + 2;
493        // splitIndex = subStr.indexOf(escapedChars[i], fromIndex);
494        // }
495        // }
496        //
497        // // escape characters for neighborhood
498        // if ((splitIndex = neighborhood.indexOf(escapedChars[i])) > -1) {
499        // if (DEBUG) {
500        // System.out.println("==== Found: " + escapedChars[i] + " in neighborhood text ====");
501        // }
502        // int fromIndex = 0;
503        // while (splitIndex > -1) { // check for mulitiple of same
504        // // escapedChar in substr
505        // // insert \ in front of the escaped character
506        // tmpStr = neighborhood.substring(0, splitIndex) + escape + neighborhood.substring(splitIndex);
507        // neighborhood = tmpStr;
508        // fromIndex = splitIndex + 2;
509        // splitIndex = neighborhood.indexOf(escapedChars[i], fromIndex);
510        // }
511        // }
512        // }
513        //
514        // if (DEBUG) {
515        // System.out.println("Processed substr: " + subStr);
516        // System.out.println("Processed neighborhood: " + neighborhood);
517        // }
518        //
519        // // create regex for substr
520        // String[] toks = subStr.split(" ");
521        // String regex_substr = "";
522        // for (int i = 0; i < toks.length; i++) {
523        // // if (escapedChars.indexOf(toks[i]) > -1) {
524        // // regex += "\\";
525        // // }
526        // regex_substr += toks[i];
527        // if (i < toks.length - 1) {
528        // regex_substr += "\\s*";
529        // } else {
530        // regex_substr += "";
531        // }
532        // }
533        //
534        // // create regex for neighborhood
535        // toks = neighborhood.split(" ");
536        // String regex_neigh = "";
537        // for (int i = 0; i < toks.length; i++) {
538        // // if (escapedChars.indexOf(toks[i]) > -1) {
539        // // regex += "\\";
540        // // }
541        // regex_neigh += toks[i];
542        // if (i < toks.length - 1) {
543        // regex_neigh += "\\s*";
544        // } else {
545        // regex_neigh += "";
546        // }
547        // }
548        //
549        // if (DEBUG) {
550        // System.out.println("REGEX_SUBSTR:" + regex_substr);
551        // System.out.println("REGEX_NEIGH:" + regex_neigh);
552        // System.out.println("WHOLESTR: " + wholeStr);
553        // }
554        //
555        // Pattern neighPattern = Pattern.compile(regex_neigh);
556        // Matcher neighMatcher = neighPattern.matcher(wholeStr);
557        //
558        // if (neighMatcher.find(startLookingAtIndex)) {
559        // if (DEBUG) {
560        // System.out.println("FOUND NEIGHBORHOOD IN WHOLESTR");
561        // }
562        // Pattern subStrPattern = Pattern.compile(regex_substr);
563        // Matcher subStrMatcher = subStrPattern.matcher(neighMatcher.group());
564        // int offset = neighMatcher.start();
565        // if (DEBUG) {
566        // System.out.println("FOUND SUBSTRING IN NEIGHBORHOOD");
567        // }
568        // if (subStrMatcher.find()) {
569        //                
570        // span[0] = Integer.toString(subStrMatcher.start() + offset);
571        // span[1] = Integer.toString(subStrMatcher.end() + offset);
572        // span[2] = subStrMatcher.group();
573        // }
574        // }
575        //
576        // return span;
577        // }
578    
579        /**
580         * Taken from: http://userpage.fu-berlin.de/~ram/pub/pub_jf47hxhHHt/java_sax_parser_en
581         * 
582         * @return an XMLReader
583         * @throws Exception
584         */
585    
586        final public static XMLReader makeXMLReader() throws Exception {
587            final SAXParserFactory saxParserFactory = SAXParserFactory.newInstance();
588            final SAXParser saxParser = saxParserFactory.newSAXParser();
589            final XMLReader parser = saxParser.getXMLReader();
590            return parser;
591        }
592    
593        /**
594         * Replaces XML special characters with their ASCII equivalents &amp; --> &, etc.
595         * 
596         * @param line
597         *            text to be converted
598         * @return the converted text
599         */
600        public static String replaceXMLspecialCharacters(String line) {
601            // # &amp;
602            // # < - &lt;
603            // # > - &gt;
604            // # " - &quot;
605            // # ' - &#39;
606            line = line.replaceAll("&amp;", "&");
607            line = line.replaceAll("&lt;", "<");
608            line = line.replaceAll("&gt;", ">");
609            line = line.replaceAll("&quot;", "\"");
610            line = line.replaceAll("&#39", "'");
611            return line;
612        }
613    
614        /**
615         * Returns a HashMap containing the linguistic annotation tags used by the Penn Treebank, and their corresponding clause, phrase, or token labels.<br>
616         * Key = Tag, e.g. "NP"; Value = "clause" "phrase" or "token"
617         * 
618         * @return a HashMap containing tag/label pairs where the tags are Penn Treebank linguistic annotation tags and the labels are
619         *         "clause","phrase",or "token"
620         */
621        public static HashMap<String, String> getPennTreebankTagLabelLookup() {
622            HashMap<String, String> tagLabelsHash = new HashMap<String, String>();
623            tagLabelsHash.put("ROOT", ClassMentionTypes.CLAUSE);
624            tagLabelsHash.put("S", ClassMentionTypes.CLAUSE);
625            tagLabelsHash.put("SBAR", ClassMentionTypes.CLAUSE);
626            tagLabelsHash.put("SBARQ", ClassMentionTypes.CLAUSE);
627            tagLabelsHash.put("SINV", ClassMentionTypes.CLAUSE);
628            tagLabelsHash.put("SQ", ClassMentionTypes.CLAUSE);
629            tagLabelsHash.put("ADJP", ClassMentionTypes.PHRASE);
630            tagLabelsHash.put("ADVP", ClassMentionTypes.PHRASE);
631            tagLabelsHash.put("CONJP", ClassMentionTypes.PHRASE);
632            tagLabelsHash.put("FRAG", ClassMentionTypes.PHRASE);
633            tagLabelsHash.put("INTJ", ClassMentionTypes.PHRASE);
634            tagLabelsHash.put("LST", ClassMentionTypes.PHRASE);
635            tagLabelsHash.put("NAC", ClassMentionTypes.PHRASE);
636            tagLabelsHash.put("NP", ClassMentionTypes.PHRASE);
637            tagLabelsHash.put("NX", ClassMentionTypes.PHRASE);
638            tagLabelsHash.put("PP", ClassMentionTypes.PHRASE);
639            tagLabelsHash.put("PRN", ClassMentionTypes.PHRASE);
640            tagLabelsHash.put("PRT", ClassMentionTypes.PHRASE);
641            tagLabelsHash.put("QP", ClassMentionTypes.PHRASE);
642            tagLabelsHash.put("RRC", ClassMentionTypes.PHRASE);
643            tagLabelsHash.put("UCP", ClassMentionTypes.PHRASE);
644            tagLabelsHash.put("VP", ClassMentionTypes.PHRASE);
645            tagLabelsHash.put("WHADJP", ClassMentionTypes.PHRASE);
646            tagLabelsHash.put("WHADVP", ClassMentionTypes.PHRASE);
647            tagLabelsHash.put("WHNP", ClassMentionTypes.PHRASE);
648            tagLabelsHash.put("WHPP", ClassMentionTypes.PHRASE);
649            tagLabelsHash.put("X", ClassMentionTypes.PHRASE);
650            tagLabelsHash.put("CC", ClassMentionTypes.TOKEN);
651            tagLabelsHash.put("CD", ClassMentionTypes.TOKEN);
652            tagLabelsHash.put("DT", ClassMentionTypes.TOKEN);
653            tagLabelsHash.put("EX", ClassMentionTypes.TOKEN);
654            tagLabelsHash.put("FW", ClassMentionTypes.TOKEN);
655            tagLabelsHash.put("IN", ClassMentionTypes.TOKEN);
656            tagLabelsHash.put("JJ", ClassMentionTypes.TOKEN);
657            tagLabelsHash.put("JJR", ClassMentionTypes.TOKEN);
658            tagLabelsHash.put("JJS", ClassMentionTypes.TOKEN);
659            tagLabelsHash.put("LS", ClassMentionTypes.TOKEN);
660            tagLabelsHash.put("MD", ClassMentionTypes.TOKEN);
661            tagLabelsHash.put("NN", ClassMentionTypes.TOKEN);
662            tagLabelsHash.put("NNS", ClassMentionTypes.TOKEN);
663            tagLabelsHash.put("NNP", ClassMentionTypes.TOKEN);
664            tagLabelsHash.put("NNPS", ClassMentionTypes.TOKEN);
665            tagLabelsHash.put("PDT", ClassMentionTypes.TOKEN);
666            tagLabelsHash.put("POS", ClassMentionTypes.TOKEN);
667            tagLabelsHash.put("PRP", ClassMentionTypes.TOKEN);
668            tagLabelsHash.put("PRP$", ClassMentionTypes.TOKEN);
669            tagLabelsHash.put("RB", ClassMentionTypes.TOKEN);
670            tagLabelsHash.put("RBR", ClassMentionTypes.TOKEN);
671            tagLabelsHash.put("RBS", ClassMentionTypes.TOKEN);
672            tagLabelsHash.put("RP", ClassMentionTypes.TOKEN);
673            tagLabelsHash.put("SYM", ClassMentionTypes.TOKEN);
674            tagLabelsHash.put("TO", ClassMentionTypes.TOKEN);
675            tagLabelsHash.put("UH", ClassMentionTypes.TOKEN);
676            tagLabelsHash.put("VB", ClassMentionTypes.TOKEN);
677            tagLabelsHash.put("VBD", ClassMentionTypes.TOKEN);
678            tagLabelsHash.put("VBG", ClassMentionTypes.TOKEN);
679            tagLabelsHash.put("VBN", ClassMentionTypes.TOKEN);
680            tagLabelsHash.put("VBP", ClassMentionTypes.TOKEN);
681            tagLabelsHash.put("VBZ", ClassMentionTypes.TOKEN);
682            tagLabelsHash.put("WDT", ClassMentionTypes.TOKEN);
683            tagLabelsHash.put("WP", ClassMentionTypes.TOKEN);
684            tagLabelsHash.put("WP$", ClassMentionTypes.TOKEN);
685            tagLabelsHash.put("WRB", ClassMentionTypes.TOKEN);
686    
687            return tagLabelsHash;
688        }
689    
690        /**
691         * This method simple returns text annotations created from finding the target string within the original text string using regular expressions
692         * 
693         * @param target
694         * @param originalText
695         * @param startLookingForMatchesAtIndex
696         *            the index where we will start looking for matches within the original text
697         * @param documentID
698         *            this is optional. If one if provided it will be used, if null is provided, -1 will be used.
699         * @param classMentionType
700         *            this is also optional. If one is provided, a ClassMention of that type will be added to the TextAnnotation
701         * @param annotator
702         *            this is also options. If one is provided, it is added to the TextAnnotation. If it is null, a default annotator is added instead.
703         * @return
704         */
705        public static List<TextAnnotation> findTargetInText(String targetText, String originalText, int startLookingForMatchesAtIndex,
706                String documentID, String classMentionType, Annotator annotator, boolean unconstrained) {
707            List<TextAnnotation> targetAnnotationsToReturn = new ArrayList<TextAnnotation>();
708    
709            /* Check for a null document ID. If one has not been provided, then just assign "-1" */
710            if (documentID == null) {
711                documentID = "-1";
712            }
713    
714            try {
715                /*
716                 * Convert targetText into pattern by making spaces optional. We do this primarily for the KeX gene finder which tokenizes parentheses. If
717                 * spaces are not made optional, then the target text will not be found with the regex.
718                 */
719                /* get the length of the target text before adding escapes for special characters */
720                int targetTextLength = targetText.length();
721                targetText = GenericTextUtils.escapeCharacterForRegEx(targetText);
722                targetText = targetText.replaceAll(" ", "\\\\s?");
723    
724                if (DEBUG) {
725                    System.out.println("DEBUG: GenericTextUtils -- TargetText Pattern: " + targetText + " start_looking_index: "
726                            + startLookingForMatchesAtIndex);
727                }
728                /* Initialize the pattern to search for the targetText within the originalText */
729                Pattern pattern;
730                /*
731                 * if the pattern is a word, we want to use the pattern below so that we don't find words inside of words, however, if it is a symbol e.g. / ( ),
732                 * etc. then we want to be able to find the symbol in between two words e.g. p53/p35. We will make the assumption here that if the target
733                 * is only 1 character wide, then we will search for it anywhere, but in a case sensitive manner.
734                 * 
735                 * if the target text is a number, then we use the less constrained search as well
736                 */
737                if (unconstrained) {
738                    pattern = Pattern.compile("(.*?)(" + targetText + ")(.*?)");
739                } else {
740    
741                    if (targetTextLength > 1 && !isNumber(targetText)) {
742                        // pattern = Pattern.compile("(^|[^a-z])(" + targetText + ")($|[^a-z])", Pattern.CASE_INSENSITIVE);
743                        // pattern = Pattern.compile("(^|[\\W])(" + targetText + ")($|[\\W])");
744                        pattern = Pattern.compile("(^|[^a-zA-Z])(" + targetText + ")($|[^a-zA-Z])");
745                    } else {
746                        pattern = Pattern.compile("(.*?)(" + targetText + ")(.*?)");
747                    }
748                }
749                // pattern = Pattern.compile("(^|.*?)(" + targetText + ")($|.*?)");
750    
751                Matcher matcher = pattern.matcher(originalText);
752                while (matcher.find(startLookingForMatchesAtIndex)) {
753                    // System.err.println("Creating new text annotation...: " + targetText + ";;");
754                    TextAnnotation ta = new TextAnnotation();
755                    ta.setDocumentID(documentID);
756                    ta.setAnnotationSpanEnd(matcher.end(2));
757                    ta.setAnnotationSpanStart(matcher.start(2));
758                    ta.setCoveredText(originalText.substring(ta.getAnnotationSpanStart(), ta.getAnnotationSpanEnd()));
759    
760                    // /* adjust spans if there are spaces at the end of the annotation */
761                    // while (ta.getCoveredText().endsWith(" ")) {
762                    // ta.setAnnotationSpanEnd(ta.getAnnotationSpanEnd()-1);
763                    // ta.setCoveredText(originalText.substring(ta.getAnnotationSpanStart(), ta.getAnnotationSpanEnd()));
764                    // }
765    
766                    if (annotator != null) {
767                        ta.setAnnotator(annotator);
768                    } else {
769                        ta.setAnnotator(new Annotator(new Integer(-1), "", "", ""));
770                    }
771    
772                    if (classMentionType != null) {
773                        ClassMention cm = new ClassMention();
774                        cm.setMentionName(classMentionType);
775                        ta.setClassMention(cm);
776                    }
777    
778                    final AnnotationSet annotationSet = new AnnotationSet(-1, "Default", "Default");
779                    ta.addAnnotationSet(annotationSet);
780    
781                    /* add the newly created TextAnnotation to the list to be returned */
782                    targetAnnotationsToReturn.add(ta);
783    
784                    if (DEBUG) {
785                        // System.out.print("DEBUG GenericTextUtils -- ");
786                        // ta.printAnnotation(System.out);
787                        System.out.println("DEBUG GenericTextUtils -- docID:" + documentID + " matched:" + matcher.group(2) + " ["
788                                + matcher.start(2) + ".." + matcher.end(2) + "]" + " text before it:" + matcher.group());
789                    }
790    
791                    /* we must update the startLookingForMatchesAtIndex variable, or else we end up in an infinite loop. */
792                    startLookingForMatchesAtIndex = ta.getAnnotationSpanEnd();
793                }
794            } catch (PatternSyntaxException pse) {
795                pse.printStackTrace();
796            }
797    
798            return targetAnnotationsToReturn;
799        }
800    
801        public static boolean isNumber(String possibleNumber) {
802            try {
803                Double d = new Double(possibleNumber);
804            } catch (NumberFormatException npe) {
805                return false;
806            }
807            return true;
808        }
809    
810        public static int findFirstNonHTMLCharacter(String htmlText) {
811    
812            // System.err.println("FINDING FIRST HTML: " + htmlText);
813            /* return -1 if text is completely HTML tags */
814            String testForAllHTML = htmlText.replaceAll("<.*?>", "").trim();
815            if (testForAllHTML.length() == 0) {
816                return -1;
817            }
818    
819            int removedCharsCount = 0;
820            /* Is the first non-space character a "<" */
821            while (htmlText.trim().charAt(0) == '<') {
822                int gtIndex = htmlText.indexOf('>') + 1;
823                removedCharsCount += gtIndex;
824                htmlText = htmlText.substring(gtIndex);
825                // System.out.println("REMOVED... " + htmlText);
826                /* remove any leading spaces of &nbsp; */
827                while (htmlText.startsWith(" ") | htmlText.startsWith("&nbsp;")) {
828                    if (htmlText.startsWith(" ")) {
829                        htmlText = htmlText.substring(1);
830                        removedCharsCount++;
831                    } else {
832                        htmlText = htmlText.substring(6);
833                        removedCharsCount += 6;
834                    }
835                }
836                if (htmlText.length() == 0) {
837                    return -1;
838                }
839            }
840    
841            return removedCharsCount;
842        }
843    
844        public static int findLastNonHTMLCharacter(String htmlText) {
845    
846            int originalLength = htmlText.length();
847    
848            /* return -1 if text is completely HTML tags */
849            String testForAllHTML = htmlText.replaceAll("<.*?>", "").trim();
850            if (testForAllHTML.length() == 0) {
851                return -1;
852            }
853    
854            int removedCharsCount = 0;
855    
856            /* Is the last non-space character a "<" */
857            int htmlTextLength = htmlText.length();
858            // System.out.println("CHECKING TEXT: Length= " + htmlTextLength + ": " + htmlText + ";;");
859            /* remove any trailing spaces or &nbsp; */
860            while (htmlText.endsWith(" ") | htmlText.endsWith("&nbsp;")) {
861                if (htmlText.endsWith(" ")) {
862                    htmlText = htmlText.substring(0, htmlTextLength - 1);
863                    removedCharsCount++;
864                } else {
865                    htmlText = htmlText.substring(0, htmlTextLength - 6);
866                    removedCharsCount += 6;
867                }
868                htmlTextLength = htmlText.length();
869                if (htmlTextLength == 0) {
870                    return -1;
871                }
872            }
873    
874            while (htmlText.charAt(htmlTextLength - 1) == '>') {
875                int ltIndex = htmlText.lastIndexOf('<');
876                removedCharsCount += (htmlTextLength - ltIndex);
877                htmlText = htmlText.substring(0, ltIndex);
878                htmlTextLength = htmlText.length();
879    
880                /* remove any trailing spaces or &nbsp; */
881                while (htmlText.endsWith(" ") | htmlText.endsWith("&nbsp;")) {
882                    if (htmlText.endsWith(" ")) {
883                        htmlText = htmlText.substring(0, htmlTextLength - 1);
884                        removedCharsCount++;
885                    } else {
886                        htmlText = htmlText.substring(0, htmlTextLength - 6);
887                        removedCharsCount += 6;
888                    }
889                    htmlTextLength = htmlText.length();
890                }
891                // System.out.println("REMOVED... " + htmlText);
892            }
893    
894            return originalLength - removedCharsCount;
895        }
896    
897        // /* replace all newline characters */
898        // htmlText = htmlText.replaceAll("\\n", "");
899        // htmlText = htmlText.replaceAll("\\r", "");
900        //        
901        // String patternStr;
902        //        
903        // if (reversed) {
904        // patternStr = ">.*?<";
905        // } else {
906        // patternStr = "<.*?>";
907        // }
908        //        
909        // String testForAllHTML = htmlText.replaceAll(patternStr, "").trim();
910        // if (testForAllHTML.length() == 0) {
911        // return -1;
912        // }
913        //        
914        // Pattern p = Pattern.compile("(" + patternStr + ")");
915        // Matcher matcher;
916        //        
917        // System.out.println("LOOKING FOR HTML TAGS... PATTERN=" + patternStr + " Text= " + htmlText);
918        //        
919        // /* remove html tags */
920        // int removedCharsCount = 0;
921        // while (htmlText.trim().matches("^(" + patternStr + ")")) {
922        // System.out.println("HAS HTML TAG...");
923        // matcher = p.matcher(htmlText);
924        // if (matcher.find()) {
925        // removedCharsCount += matcher.end(1);
926        // htmlText = htmlText.substring(matcher.end(1));
927        // System.out.println("REMOVED HTML TAG: " + htmlText);
928        // } else {
929        // System.err.println("WARNING -- HTMLParagraphProcessor: Expected matcher to match...");
930        // }
931        // }
932        //        
933        // /* remove any leading spaces */
934        // while (htmlText.charAt(0) == ' ') {
935        // htmlText = htmlText.substring(1);
936        // removedCharsCount++;
937        // }
938        //        
939        // return removedCharsCount;
940        // }
941    
942        // public static int findLastNonHTMLCharacter(String htmlText) {
943        //        
944        // StringBuilder sb = new StringBuilder(htmlText);
945        // htmlText = sb.reverse().toString();
946        // //System.out.println("REVERSED: " + htmlText);
947        // int value = findFirstNonHTMLCharacter(htmlText, true);
948        // if (value == -1) {
949        // return -1;
950        // } else {
951        // return htmlText.length() - value;
952        // }
953        //    
954        // // Pattern p = Pattern.compile("(<.*?>)$");
955        // // Matcher matcher;
956        // //
957        // // /* remove html tags */
958        // // int removedCharsCount = 0;
959        // // while (htmlText.trim().matches("<.*?>$")) {
960        // // matcher = p.matcher(htmlText);
961        // // if (matcher.find()) {
962        // // removedCharsCount += (htmlText.length() - matcher.start(1));
963        // // htmlText = htmlText.substring(0,matcher.start(1));
964        // // System.out.println("MATCHED: " + matcher.group(1) + "[" + matcher.start(1)+ ".." + matcher.end(1) + "]");
965        // // System.out.println("REMOVED: " + htmlText);
966        // //
967        // // /* remove any trailing spaces */
968        // //
969        // // int htmlTextLength = htmlText.length();
970        // // while (htmlText.charAt(htmlTextLength -1) == ' ') {
971        // // htmlText = htmlText.substring(0, htmlText.length() -1);
972        // // htmlTextLength = htmlText.length();
973        // // removedCharsCount++;
974        // // }
975        // //
976        // // } else {
977        // // System.err.println("WARNING -- HTMLParagraphProcessor: Expected matcher to match...");
978        // // }
979        // // }
980        // //
981        // //
982        // //
983        // // return removedCharsCount;
984        // }
985    
986        // public static void main(String[] args) {
987        // String htmlText = "This research begins with the premise, pioneered by Block et<SUP> </SUP>al. (1<A HREF=\"#B1\"><IMG BORDER=1
988        // WIDTH=8 HEIGHT=7 ALT=\"Go\" SRC=\"/icons/fig-down.gif\"></A>), that using nationally representative dietary data in<SUP> </SUP>a
989        // systematic way to create an FFQ nutrient database will provide<SUP> </SUP>the most unbiased nutrient estimates for an FFQ to be used
990        // among<SUP> </SUP>US adults. This reasoning is based on the premise that non-data-driven<SUP> </SUP>methods will necessarily involve
991        // more extensive judgments and<SUP> </SUP>assumptions on the part of investigators that may not accurately<SUP> </SUP>represent current
992        // food consumption and composition and that<SUP> </SUP>are not reproducible across investigators. Although all methods<SUP>
993        // </SUP>require some degree of judgment and decision making (such as<SUP> </SUP>in the food grouping step), the more this is minimized,
994        // the<SUP> </SUP>more likely that the instruments will reflect the reality of<SUP> </SUP>consumption in the population.<SUP> </SUP>";
995        //        
996        // int firstNonHtmlChar = GenericTextUtils.findFirstNonHTMLCharacter(htmlText);
997        // System.out.println(htmlText.substring(firstNonHtmlChar));
998        //        
999        // htmlText = "<A HREF=\"blah\">This research begins with the premise, pioneered by Block et<SUP> </SUP>al. (1<A HREF=\"#B1\"><IMG
1000        // BORDER=1 WIDTH=8 HEIGHT=7 ALT=\"Go\" SRC=\"/icons/fig-down.gif\"></A>), that using nationally representative dietary data in<SUP>
1001        // </SUP>a systematic way to create an FFQ nutrient database will provide<SUP> </SUP>the most unbiased nutrient estimates for an FFQ to
1002        // be used among<SUP> </SUP>US adults. This reasoning is based on the premise that non-data-driven<SUP> </SUP>methods will necessarily
1003        // involve more extensive judgments and<SUP> </SUP>assumptions on the part of investigators that may not accurately<SUP> </SUP>represent
1004        // current food consumption and composition and that<SUP> </SUP>are not reproducible across investigators. Although all methods<SUP>
1005        // </SUP>require some degree of judgment and decision making (such as<SUP> </SUP>in the food grouping step), the more this is minimized,
1006        // the<SUP> </SUP>more likely that the instruments will reflect the reality of<SUP> </SUP>consumption in the population.<SUP> </SUP>";
1007        // firstNonHtmlChar = GenericTextUtils.findFirstNonHTMLCharacter(htmlText);
1008        // System.out.println(htmlText.substring(firstNonHtmlChar));
1009        //        
1010        //        
1011        // htmlText = "<A HREF=\"blah\"> <A HREF=\"blah\"> <A HREF=\"blah\"><A HREF=\"blah\"> <A HREF=\"blah\"> This research begins with the
1012        // premise, pioneered by Block et<SUP> </SUP>al. (1<A HREF=\"#B1\"><IMG BORDER=1 WIDTH=8 HEIGHT=7 ALT=\"Go\"
1013        // SRC=\"/icons/fig-down.gif\"></A>), that using nationally representative dietary data in<SUP> </SUP>a systematic way to create an FFQ
1014        // nutrient database will provide<SUP> </SUP>the most unbiased nutrient estimates for an FFQ to be used among<SUP> </SUP>US adults. This
1015        // reasoning is based on the premise that non-data-driven<SUP> </SUP>methods will necessarily involve more extensive judgments and<SUP>
1016        // </SUP>assumptions on the part of investigators that may not accurately<SUP> </SUP>represent current food consumption and composition
1017        // and that<SUP> </SUP>are not reproducible across investigators. Although all methods<SUP> </SUP>require some degree of judgment and
1018        // decision making (such as<SUP> </SUP>in the food grouping step), the more this is minimized, the<SUP> </SUP>more likely that the
1019        // instruments will reflect the reality of<SUP> </SUP>consumption in the population.<SUP> </SUP>";
1020        // firstNonHtmlChar = GenericTextUtils.findFirstNonHTMLCharacter(htmlText);
1021        // System.out.println(htmlText.substring(firstNonHtmlChar));
1022        // }
1023    
1024        // public static void main(String[] args) {
1025        // System.out.println("INPUT DOC TEXT LENGTH: " + args[1].length());
1026        // ArrayList ar = GenericTextUtils.tokenizeIntoSentences(args[0], args[1]);
1027        // ArrayList sentences = (ArrayList) ar.get(0);
1028        // ArrayList words = (ArrayList) ar.get(1);
1029        //
1030        // for (Iterator iter = sentences.iterator(); iter.hasNext();) {
1031        // String[] sent_span = (String[]) iter.next();
1032        // System.out.println("SENTENCE: " + sent_span[0] + " -- " + sent_span[1] + "\t" + sent_span[2]);
1033        // }
1034        //
1035        // for (Iterator iter = words.iterator(); iter.hasNext();) {
1036        // String[] word_span = (String[]) iter.next();
1037        // System.out.println("WORD: " + word_span[0] + " -- " + word_span[1] + "\t" + word_span[2]);
1038        //
1039        // }
1040        //
1041        // }
1042    
1043        public static String getTokensBefore(int numTokens, int index, String documentText) {
1044            StringBuilder sb = new StringBuilder(documentText);
1045            String reverseDocText = sb.reverse().toString();
1046            int revIndex = documentText.length() - index;
1047    
1048            String revNextToken = getTokensAfter(numTokens, revIndex, reverseDocText);
1049            sb = new StringBuilder(revNextToken);
1050            return sb.reverse().toString();
1051        }
1052    
1053        public static String getTokensAfter(int numTokens, int index, String documentText) {
1054            int nextSpaceIndex = index;
1055            for (int i = 0; i < numTokens; i++) {
1056                nextSpaceIndex = documentText.indexOf(" ", nextSpaceIndex + 1);
1057                if (nextSpaceIndex == -1) {
1058                    break;
1059                }
1060            }
1061            if (nextSpaceIndex == -1) {
1062                if (index < documentText.length()) {
1063                    /* return the end of the document text */
1064                    return documentText.substring(index, documentText.length());
1065                } else {
1066                    return "";
1067                }
1068            } else {
1069                return documentText.substring(index, nextSpaceIndex);
1070            }
1071        }
1072    
1073    }
```
